# Brain abscesses and intracranial empyema due to dental pathogens: Case series

**DOI:** 10.1016/j.ijscr.2020.03.025

**Published:** 2020-03-28

**Authors:** Charlène Kichenbrand, Alix Marchal, Alizée Mouraret, Nasr Hasnaoui, Julie Guillet, Fabien Rech, Berengère Phulpin

**Affiliations:** a Oral Surgery Department, Faculty of Odontology, Lorraine University, 96 Avenue Maréchal De Lattre De Tassigny, 54004 Nancy, France; b Odontology Department, Regional University Hospital, 5 Rue Du Morvan, 54500 Vandoeuvre-lès-Nancy, France; c Neurosurgery Department, Regional University Hospital, 29 Avenue Maréchal De Lattre De Tassigny, 54035 Nancy, France

**Keywords:** Brain abscess, Intracranial empyema, Oral infection, Streptococci, Odontogenic, Teeth

## Abstract

**Introduction:**

Spontaneous brain abscess and intracranial empyema are rare but life-threatening diseases. They can result from potential complications of oral chronic infections. Given the similarity between most of the germs commonly found both in cerebral abscess and in oral cavity and its anatomic proximity, the oral cavity represents a major source of cerebral infections.

**Presentation of cases:**

We report here a series of seven cases of intracranial abscesses and empyema due to dental pathogens that occurred in the neurosurgery department of our institute. Five of those seven cases were then cared in the oral surgery department of our institute.

**Discussion:**

Bacteriological examination of the brain abscesses identified an oral commensal bacterium belonging to streptococci group in six patients (85.7%) and *Porphyromonas Gingivalis*, a pathogen oral germ involved in periodontal diseases in the seventh patient. For all patients examined, oral status and oral health were poor or moderate with the presence of dental caries, missing teeth and abundant dental plaque. Except the oral cavity, no other front door or infectious site were found.

**Conclusion:**

This rare series of cases highlights the importance of systematically explore the oral cavity in presence of intracranial infection and to radically eradicate the potential infectious foci.

## Highlights

• Cerebral infection is rare but life-threatening disease.
• Dental origin for brain abscesses or intra cranial empyema is very rarely reported in the literature.
• Infected maxillary teeth are probably involved in contiguous spread of the infection to the brain.
• There is currently no guidelines in oral surgery to manage patients with cerebral infection from dental origin.

## 1 Introduction

Brain abscesses and intracranial empyema are both focal suppurative infections of the brain, respectively intraparenchymatous and sub/extradural. Despite the recent therapeutic progresses including antibiotic therapy, both are still life-threatening pathologies and can lead to severe sequelae as moderate or severe incapacity [1]. Limited data are available on the epidemiology of brain abscesses [2] but its incidence is estimated at 0.3–0.9 per 100,000 habitants in developed countries [3,4]. Mortality stand between 17% and 37% [5]. On average, less than a half of the patients fully recover and a third keeps mild deficits [[5], [6], [7]]. Brain focal infection can occur after traumatic brain or head injury or neurosurgery (10% of cases [8]). It can also be secondary to the spread of pathogens from septic focus elsewhere, either by contiguous extension or systemic circulation. The bacteria spread can occur on systemic infection by haematogenous diffusion (30% cases [4]). However, the main aetiology of brain abscesses is the diffusion of contiguous infection (40–50% of cases) [4,8,10]. Mostly, it comes due to otitis media, frontal sinusitis, or oral infection [13]. Finally, there is no etiology clearly identified in 10–20 per cent of cases [4,8,11].

The most commonly bacterial species involved are streptococci (54%), staphylococci (15%) and anaerobic bacteria (17%) [3,11].

A dental origin could explain the majority of brain abscesses without obvious front door [9,12,14]. Oral cavity pathogens may reach the brain in various ways: 1) direct contiguity spread (notably upper jaw teeth), 2) systemic hematogenous bacteremia (after invasive oral procedure or even spontaneous), 3) direct veinous drainage. Cerebral abscesses linked to oral infection usually are polymicrobial [8]: streptococci, staphylococci, *Actinomyces sp*, *Actinobacillus sp*, *Fusobacterium sp*. The *staphylococcus* species are not commensals in the oral cavity but are found in acute oral and cervicofacial infectious diseases as cellulitis. Streptococci, *Actinomyces sp and Fusobacterium sp* are endemics in the oral cavity and are involved in most of dental diseases (endodontic pathologies, periodontal diseases, abscesses). They are also found in large quantity in the dental plaque, which presence indicates poor level of oral hygiene.

Only a very limited number of cases of brain abscesses due to dental pathogens have been reported and documented in contemporary literature [12].

We report here a series of seven cases of brain abscess or empyema caused by dental germs followed in the neurosurgery or oral surgery departments of our institute between 2014 and 2018.

## 2 Presentation of cases

A close collaboration between neurosurgery department and oral surgery department of our institute allowed to identify patients presenting a brain abscess or intracranial empyema due to dental germ, between 2014 and 2018. We screened seven patients for whom obvious infectious site were excluded (pulmonary, cardiac, cutaneous, urogenital) and infected with pathogens consistent with oral origin.

The gender ratio was five males for two females. The mean age of women was 58 (range 57–58 years) and 44 for the men (range 24–57 years). More than half of the patients (n = 4, 57%) presented predisposing factors (congenital heart disease (n = 1), diabetes mellitus (n = 1), pansinusitis (n = 2)) and none for the other three. Headache was the most common single presenting symptom that occurred in 4 (57%) patients. Three patients (42.9%) suffered from neurological deficit.

**Fig. 1:**
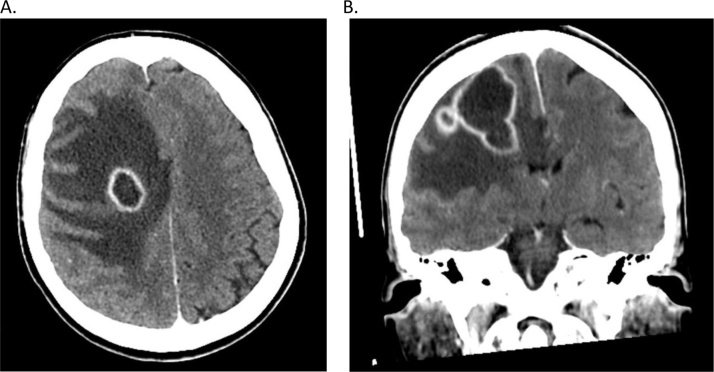
Initial Computerized tomography showing right frontal abscess in case “1” A. Axial section B. Frontal section.

CT-scan or MRI detected frontal (42.9%, n = 3) (Fig. 1), parietal (14.3%, n = 1), occipital (14.3%, n = 1) or cerebellar lesion (28.6%, n = 2). Evolution was fatal in two cases (28.6%), three patients fully recovered (42.9%) and two patients still have neurological deficit (28.6%) (Table 1). Bacterial samplings and microbiological investigations found single (42.9%, n = 3) or multiple (57.1%, n = 4) pathogens in the infection lesion. With one exception, isolated pathogens belong to streptococci group (85.7%, n = 6), including *Streptococcus Intermedius* (66.7% (n = 4)), *Parvimonas Micra* (16.7%, n = 1), *Streptococcus Constellatus* (33.3%, n = 2), *Streptococcus Anginus* (16.7%, n = 1), *Streptococcus Gordonii* (16.7%, n = 1). In one case, a gram-negative anaerobe, *Porphyromonas Gingivalis*, was identified (14.3%, n = 1).

**Table 1:** Age, gender, medical history, lesion, clinical presentation and outcome.

Case	Age	Gender	Comorbidities	Localization of the lesion	Type of lesion	Clinical presentation	Outcome
1	59	F	–	Frontal	Brain abscess	Headache	Full recovery
2	57	F	Congenital heart disease	Cerebellum	Brain abscess	Headache Vertigo Asthenia	Neurological deficit
3	52	M	–	Cerebellum	Brain abscess	Cerebellar syndrome	Neurological deficit
4	37	M	–	Occipital	Brain abscess	Neurological deficit Coma	Death
5	57	M	Diabetes mellitus	Parietal	Brain abscess	Headache	Death
6	24	M	Pansinusitis	Frontal	Intracranial empyema	Headache	Full recovery
7	50	M	Maxillar and frontal sinusitis	Frontal	Intracranial empyema	Aphasia Hemiparesia	Full recovery

**Fig. 2:**
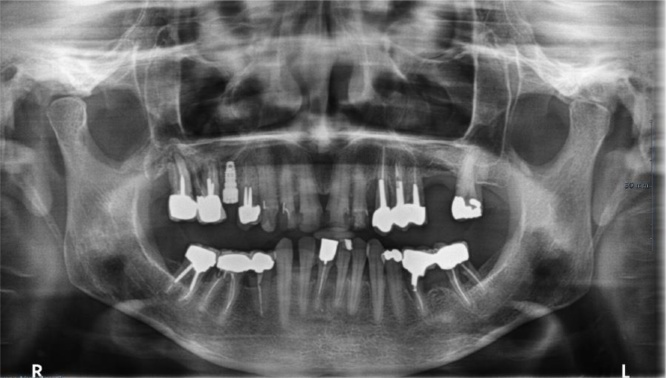
Initial orthopantomogram (case 1) showing maxillary (teeth number 14-16-17-23) or mandibular (teeth number 35-37-45-46) chronic infectious sites.

Concerning the oral status and level of oral hygiene, two patients died early after their admission and therefore could not be examined in the oral surgery department. For the other five, the oral examination provided information on oral status and hygiene. Four patients presented a very low oral status (tooth missing, dental caries, multiple crown destructions, chronic periapical diseases) and a low level of hygiene (lack of dental hygiene, abundant dental plaque) and one a quite poor oral status (periapical diseases but low amount of dental plaque). Oral radiological examination with orthopantomogram highlighted an average of 10 teeth with chronic infectious lesions in each patient (range 6–17 teeth), as illustrated by case 1 orthopantomogram (Fig. 2). We included teeth with chronic infections as periapical disease, granuloma, cyst, and endo-periodontal bone damage. Among these teeth, 80.4% were maxillary ones (range 50–100%) (Table 2).

**Table 2:** Pathogens, oral status and oral infectious foci.

s	Pathogens	Oral status and level of hygiene	Number of oral infectious sites (n) and teeth involved in	% of infected maxillary teeth
1	*Streptococcus Intermedius-Parvimonas Micra*	Middle	n = 8	50
			14/16/17/23/36/37/45/46	
2	*Streptococcus Intermedius*	Low	n = 6	100
			15/16/17/25/26/27	
3	*Streptococcus Intermedius*	Low	n = 6	83
			16/17/24/25/27/35	
4	*Porphyromonas Gingivalis*	–	–	–
	*Campylobacter Rectus*			
5	*Streptococcus Intermedius*	–	–	–
6	*Streptococcu s Constellatus*	Low	n = 17	76
	*Streptococcus Anginus*		11/12/13/14/15/16/17/21/22/23/24/27/28/36/38/44/47	
7	*Streptococcus Constellatus*	Low	n = 14	93
	*Streptococcus Gordonii*		11/12/14/15/17/18/21/22/23/25/27/28/38	

All infected teeth were removed by oral surgeons in the odontology department of our institute.

This work has been reported in line with the PROCESS criteria [15].

## 3 Discussion

Although brain abscess and empyema remain an uncommon pathology, the literature reports cases involving dental germs [9,12,14]. The mean age of our patients (48 years) and gender ratio is similar to the other series reported [16,17]. In 100% out of our cases, at least one of the symptoms of the classic triad (fever, headache, neurological default) was present, mostly the headache (57%). The outcome is also in the average of the cases found in the literature, including a mortality rate of 28.6% [1].

The oral cavity is known to constitute a reservoir of rich and varied bacterial flora. Among those bacteria, some are repeatedly depicted in cerebral abscesses and empyema as the streptococci group [11]. This in accordance with our results: *Streptococcus Intermedius* or other streptococci were found in six out of seven of our cases (85.7%). The seventh case involved *Porphyromonas Gingivalis*, an oral anaerobic gram-negative bacterium, when we know that 17% of cerebral abscesses contain anaerobic germs [3], which is also consistent with our data (14.3%). The difficulty of assessing an oral etiology for brain abscesses lies in the fact that some pathogens are not specific of the oral cavity. Nevertheless, if we add types of pathogens found in abscesses to the fact that no other infectious portal of entry was found in the cases we report here, the oral origin can be clearly established.

Interestingly, in our cases, all the patients presented a majority of maxillary chronic infected sites, that could be an argument for a contiguous dissemination between oral cavity and brain. The average of infected maxillary teeth was about 80% of the total teeth removed, as resumed in Table 2. Furthermore, the patients presented a low level or oral hygiene and poor periodontal condition. In the cases treated in our department, all patients had six teeth or more that presented chronic infections with endodontic or periodontal lesion (Table 2).

Only one of our cases (patient 2) has in his medical background a predisposing factor of systemic infection by haematogenous diffusion; congenital cyanotic heart disease. However, this patient also presented a very poor oral status with six maxillary dental chronic infectious foci that could have led to a local contiguous diffusion of pathogens.

Concerning the two patients presenting frontal subdural empyema (cases 6–7), it first occurred by maxillary then frontal sinusitis or pansinusitis. One of the patients (case 6) reported maxillary dental pain before the apparition of the sinusitis, localized on the same side. On our knowledge, there is no larges series in the literature concerning the prevalence of empyema caused by sinusitis or chronic dental disease, but it is presumed to be between 3–10% of the cases [13]. In our two cases, respectively 76% (13 out of 17 teeth) and 93% (13 out of 14 teeth) of the infected teeth were upper maxillary ones.

## 4 Conclusion

There are currently no guidelines in odonto-stomatology for the care of patients with cerebral infections. Antibiotic therapy is not recommended before or after any oral invasive procedure, nor any dental therapeutic strategies recommendations. Nevertheless, in the light of the potential serious life-threatening complications and severe sequelae incurred, a conservative approach must not be envisaged for infected teeth. Identification of the oral infected area has to be careful and systematized. Facing the emergency imposed by those pathologies, surgical procedure must be preferred to restorative one, as endodontic treatments. The oral cavity harbors abundant heterogeneous microflora that can be involved in brain abscesses. Some chronic dental conditions are predisposing to bacterial dissemination as gingivitis and periodontal diseases. In addition, it has been shown that even simple tooth brushing or chewing can induce transient bacteremia [18].

Thus, focus should be placed on the prevention and the importance to reach and maintain a high level of oral hygiene.

## Sources of funding

No funding declared

## Ethical approval

A single monocentric retrospective series of cases that reports the observation of subjects receiving the normal standard of care (no new or novel procedures) is generally not considered research and thus is exempt from needing IRB approval in France.

## Consent

Written consents obtained.

## Author contribution

Conception and design of the study, or acquisition of data: C. Kichenbrand, A. Marchal, A. Mouraret, N. Hasnaoui, J. Guillet, F. Rech, B. Phulpin. Drafting the article of revising it critically for intellectual content: C. Kichenbrand, F. Rech, B. Phulpin. Final approval of the version to be submitted: C. Kichenbrand, A. Marchal, A. Mouraret, N. Hasnaoui, J. Guillet, F. Rech, B. Phulpin.

## Registration of research studies

Study registered at at http://www.researchregistry.com

UIN: researchregistry5371.

## Guarantor

Dr Bérengère Phulpin.

Dr Fabien Rech.

## Provenance and peer review

Not commissioned, externally peer-reviewed.

## Declaration of Competing Interest

No conflicts of interest declared.
